# The palliative care experience in Irish nursing homes during the COVID-19 pandemic: a survey of residents, family, and staff

**DOI:** 10.1186/s12904-024-01458-8

**Published:** 2024-05-22

**Authors:** Owen Doody, John Lombard, Tara Delamere, Mary Rabbitte

**Affiliations:** 1 Health Research Institute, Department of Nursing and Midwifery, University of Limerick, Limerick, Ireland; 2 School of Law, Faculty of Arts, Humanities and Social Sciences, University of Limerick, Limerick, Ireland; 3 Project Manager, All Ireland Institute of Hospice and Palliative Care (AIIHPC), Dublin, Ireland; 4 Research Programme Manager, All Ireland Institute of Hospice and Palliative Care (AIIHPC), Dublin, Ireland

**Keywords:** Palliative care, COVID-19, Nursing home, Policy, Human rights, Equality

## Abstract

**Background:**

Nursing homes and other long-term care services account for a disparate share of COVID-19 cases and casualties worldwide. During COVID-19 there is a distinct need to preserve a holistic view of the wellbeing of residents of nursing homes, be mindful of their rights as citizens, and to be aware of protecting residents from infection. The delivery of health and social care throughout a pandemic must remain person-centred and adhere to a human rights-based approach.

**Methods:**

This study aimed to capture nursing home residents, their families and staff’s perspective of the nursing homes residents experience, approaches of staff and the nursing home environment. An online survey was distributed via stakeholder networks and online platforms across Ireland. This study was performed and reported in line with the Consensus-Based Checklist for Reporting of Survey Studies (CROSS).

**Results:**

25 residents, 42 family members and 51 staff completed the survey (*n* = 118). Across the domains measured all but one aspect scored above 50% **(**residents get up and go to bed when they want 41.5%) with the highest score of 89.1% scored for the nursing home is comfortable and well-kept. Results highlight evidence of positive experiences and endeavours to preserve social connections, residents were in a safe place cared for by staff who did their best in a difficult position and who went above and beyond their duty of care. However, some families reported poor communication, no internet connections, not enough phones or tablets, and that staff were busy and unable at times to assist residents who needed help using phones/tablets.

**Conclusion:**

This study highlights the importance of human rights and how they ought to inform and shape the advancement of public health advice and policy documents. Overall, nursing home residents, their families and staff reported favourably on the study measures. However, issues pertaining to communication are essential and there is a need to address issues such as the provision of accurate timely information, communication infrastructure and resources, and inconsistencies in communications. Of note is that while healthcare professionals have a duty to uphold the rights of nursing home residents, they themselves have human rights which must also be protected and supported.

**Supplementary Information:**

The online version contains supplementary material available at 10.1186/s12904-024-01458-8.

## Background

Palliative care involves the avoidance and relief of suffering and enhancement of quality of life [1]. Suffering in health is related to illness or injury of any kind and encapsulates physical, spiritual, social, and/or emotional functioning [2]. Thus, the most recent palliative care definition sees palliative care as the “active holistic care of individuals across all ages with serious health-related suffering due to severe illness, and especially of those near the end of life aiming to improve the quality of life of patients, their families and their caregivers’’ [3]. Essentially, the goal of palliative care is to achieve optimal quality of life for patients while also addressing the needs of their families [4]. Palliative care incorporates symptom management along with social, psychological, and spiritual support, involving sharing information, thereby facilitating a team approach to care between healthcare professionals and organisations and listening to patients’ preferences [5]. Advocating for these preferences is pursued where feasible, although adhering to these preferences may not always possible [5]. Access to palliative care is important to patients and families at all stages of disease trajectory (from diagnosis through death and into bereavement) and is applicable to all care settings for people with either a cancer or non-cancer diagnosis [6]. The philosophy and practice of palliative care is fundamental to the care of older persons such as those in nursing homes [7].

However, the COVID-19 pandemic had a devastating effect on nursing homes internationally and although nursing homes accommodate a low percentage of the population (0.5% USA) they accounted for approximately 25% of the documented deaths due to COVID-19 [8]. While France and Ireland reported higher rates with residents of nursing homes accounting for 50% of COVID-19 deaths [8]. During lockdown periods, nursing home residents in Ireland, similar to other countries, were unable to see their families or participate in communal meals or activities due to high levels of concern that that they would be at increased risk to the virus [9]. These concerns were heightened due to underlying chronic medical conditions, congregated living quarters, vulnerability to outbreaks of respiratory pathogens, and routine contact with staff members [10]. In Ireland there are presently 578 registered nursing homes [11] which provide approximately 32,000 residential places [12]. These nursing homes are a combination of public, private, and voluntary [13]. Nursing Homes Ireland is the national representative body for both voluntary and private nursing homes, while the Health Service Executive (HSE) has oversight of public nursing homes.

In Ireland, as was the case internationally, a wide variety of COVID-19-related actions and rules were established within a short space of time to manage the spread of the virus in nursing homes, most notable of these was the implementation of visiting restrictions [14]. Internationally, guidance on visitation was regularly updated or modified to reflect changes in infection prevention and control, COVID-19 prevalence rates, and the rollout of vaccinations [15]. At certain points, during COVID-19 waves and the onset of vaccination the restrictions were modified to permit a maximum of two named visitors, visits of a limited length could be permitted, and the usage of window and remote forms of contact were introduced [16]. Such restrictions were difficult as nursing homes are not only suppliers of healthcare but are also the home environment for the person residing there. Moreover, restrictions of this nature have a clear human rights element as they engage rights to private and family life, rights to bodily integrity, and guarantees of equality [17]. In addition to imposing visitation restrictions, Irish nursing home providers had to continually respond to an ever-embryonic policy and governing landscape as national direction was updated to affiliate with evidence and guidelines issued by the National Public Health Emergency Team for COVID-19 (NPHET) and the Government of Ireland [16]. This advice focused on staffing provisions, safeguards to curb the spread of infection, procedures for isolation, the use of personal protective equipment and COVID-19 testing [16]. The speed of guidance development and change resulted in 11 versions of the Health Protection Surveillance Centre guidance in the three months between 30 March 2020 and 3 July 2020 [12]. This required nursing home providers to quickly interpret these updates, ensure staff were aware of developments, and implement the changes throughout their facility [18].

These changes coupled with the pandemic itself placed significant strain on residents, families and staff within nursing homes which have previously reported chronic staff shortages, high levels of staff turnover and high burnout [19]. Given the human rights element related to the pandemic it is incumbent on governments and service providers to appreciate how COVID-19 has affected the day-to-day lives of those concerned through capturing their perspectives [20]. With such rapid changes due to COVID-19 restrictions and its impact on the human rights and lives of nursing home residents, staff and residents’ families, and its impact on palliative care needs it is important to capture their perspectives and how we can learn from this experience. This study aims to capture nursing home residents, their family members, and staff working in nursing homes perspective of the nursing homes residents experience, approaches of staff and the nursing home environment during the COVID-19 pandemic. It contextualises this by applying a human rights lens and considering the implications for palliative care in this environment. In this way, the article reveals key challenges and areas for action.

## Methods

### Study design

The study used a quantitative design utilising a cross sectional electronic survey of residents, families, and staff of nursing homes in Ireland.

### Research setting and access

The setting was nursing homes in Ireland and access was supported by an appointed individual from both Nursing Homes Ireland and the HSE who acted as gatekeepers for the study. Participants had to be either a resident of a nursing home, a family member of a resident of a nursing home, or a staff member working in a nursing home. The gatekeepers distributed the invitation letter, information sheet and link to the survey through their network. Some nursing homes were excluded due to their participation in other national/international studies which the gatekeepers were aware of. Furthermore, the demand effect of infection and bereavement rates in individual nursing homes led the gatekeepers to exclude some nursing homes as appropriate. In total 25 nursing homes were approached by the gatekeepers and contributed to this study.

### Sample

Participants were staff of nursing homes (*n* = 51), residents of nursing homes (*n* = 25), and residents’ family members (*n* = 42) who self-selected to participate in this study.

### Data collection

Data was gathered using Qualtrics through a questionnaire developed with stakeholders for the study addressing questions related to residents, staff, and service, from residents, families and staff perspective. Questionnaires were developed over four rounds with the steering group. The initial round involved engaging with relevant literature and policies relating to nursing and palliative care and identifying key areas for questions and agreeing with the steering group. The second round involved removing areas deemed not relevant by the steering group and adding areas the steering group identified as missing. The third round focused on listing all questions remining and added in round two to identify agreement on inclusion and grouping questions similar in nature together and grouping questions under suggested headings. The fourth and final round involved the input of a steering group in reviewing the questionnaire regarding its layout, structure, heading and questions for content validity, face validity, representativeness of questions and suitability of questions (Supplementary file 1). The questionnaire was then piloted among the steering group and nursing home representatives with regards to usability, clarity, flow, and layout.

In this study, the questionnaire wording was adapted to denote the participants (resident, family, or staff) with each of the three groups asked the same set of questions. The questionnaire consisted of four sections, section one the resident (Q1 to Q13), section two the staff (Q14 to Q26), section three the service (Q27 to Q46) and section four demographic information (Q47 to Q53), and participants were afforded the opportunity to provide additional comments in each section. A survey link and study details were distributed by the gatekeepers to the nursing homes and on social media to ensure people had an opportunity to participate in the study, provide their experiences and lessen selection bias. In addition, a hard copy format and support to complete was available if requested to support any participant with technology, vison, reading or dexterity issues.

### Data analysis

Data was analysed in SPSS (Statistical Package for Social Sciences). Descriptive statistics such as frequency and percentages were used to report distribution and summary characteristics. Open-ended questions producing qualitative data were analysed by content analysis utilising the eight steps outlined by Colorafi and Evans [21] namely, (1) developing the coding framework, (2) adding memos and codes, (3) conduct first level coding, (4) categorising codes and conducting second level coding, (5) revising and redefining codes, (6) adding memos, (7) visualising the data and (8) representing the data. This process permitted the systematic detection and organisation of patterns of meaning across the data set. This included codes, categories, and themes. The quantitative findings are described firstly followed by a summation of the qualitative findings. This study was conducted and reported in line with the Consensus-Based Checklist for Reporting of Survey Studies – CROSS (supplementary file 2).

### Ethics

The researchers adhered to national and international codes of research practice and professional standards. Participants made an informed decision to participate through the provision of a study materials consisting of an invitation, information sheet, and consent was required prior to commencing the survey as part of the online survey introductory pages.

## Results

In total, 118 surveys were completed and 31 persons (6 residents, 10 staff, 15 family members) responded to open-ended questions. This section commences with an outline of the demographic data followed by the three key areas surveyed, namely experience of the nursing home resident, the approach of staff, and the nursing home environment. The findings from the open-ended comments are also reported as part of this section.

### Demographics

**Fig. 1:**
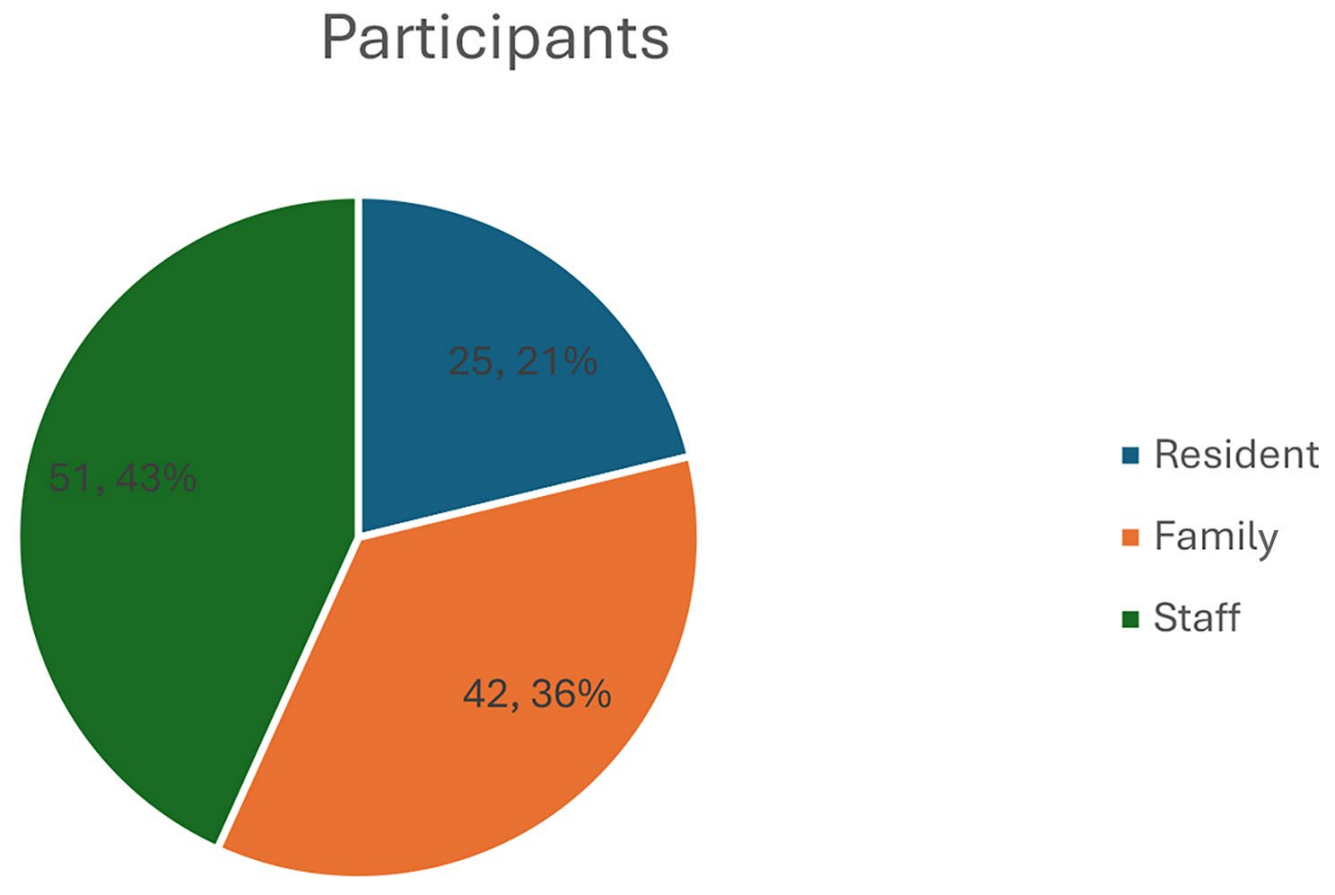
Participant demographics

**Fig. 2:**
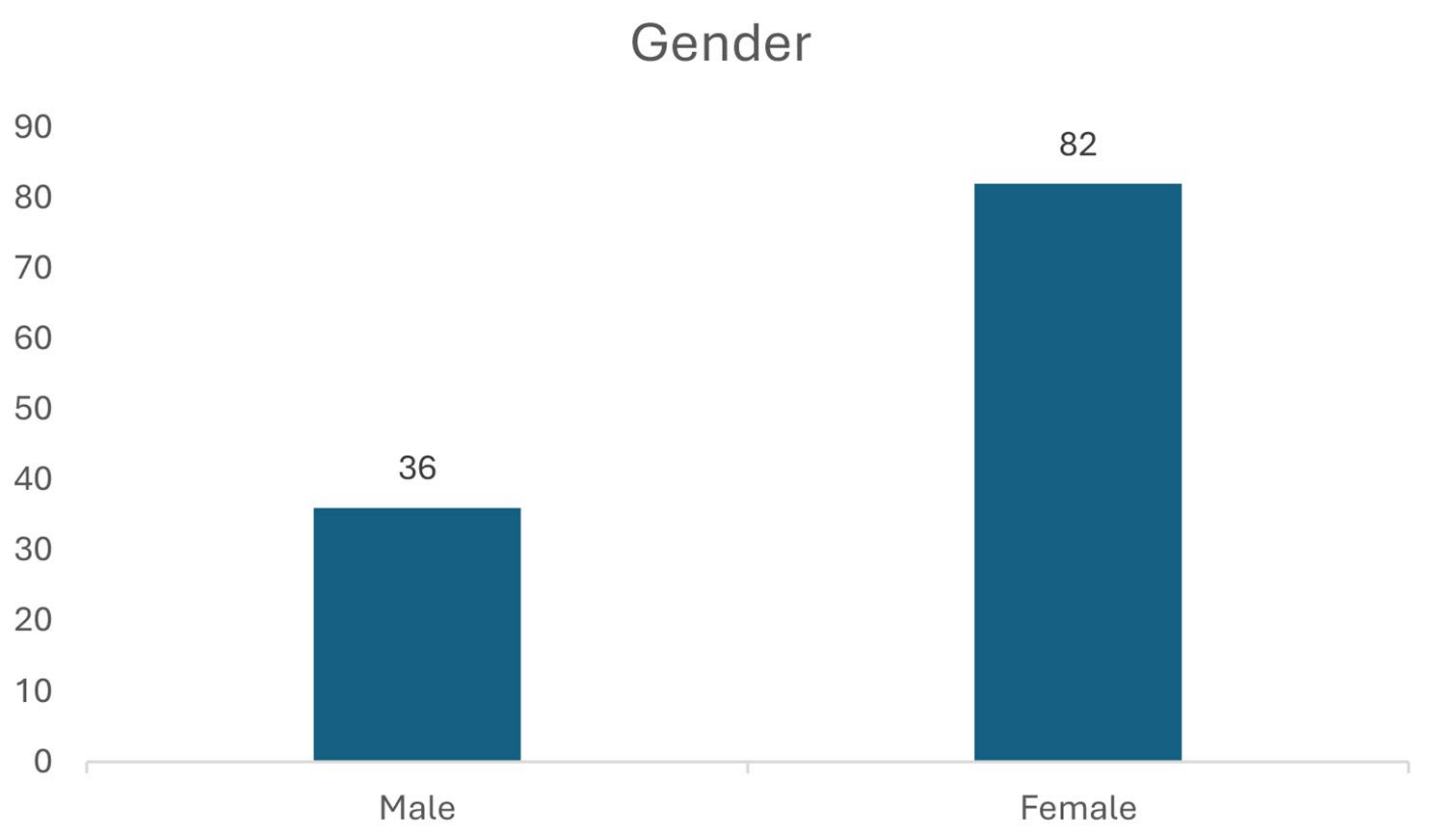
Gender

Figure 1 identifies the representation of the 118 participants and Fig. 2 gender representation. Within gender, of the residents 15 (60%) were male and 10 (40%) were females, of the family members 8 (19%) were male and 34 (81%) females, and of the staff members 13 (25.5%) were male and 38 (74.5%) females. Participant age profile ranged from 20 to 92 years with their length of stay in/or connected to a nursing home ranging from 1 to 28 years. For the resident participant group 20% (*n* = 5) resided in the nursing home for one year, 36% (*n* = 9) two years, and 44% (*n* = 11) three years. For the family participant group, 45% (*n* = 19) had a family member in the nursing home for one year, 31% (*n* = 12) two years, 5% (*n* = 2) three years, 17% (*n* = 7) four years, and 2% (*n* = 1%) ten years. Residents age profile ranged from 79 to 92 years and family age profile ranged from 30 to 91 years identifying both partners and children were represented. Overall, 83.3% (*n* = 98) participants considered that the information provided about COVID-19 was clear and understandable while 16.1% (*n* = 19) found the information provided was not clear and understandable. It is notable however that all residents (100%, *n* = 25) indicated that the information provided about COVID-19 was clear and understandable, followed by 94.1% (*n* = 48) staff, and 61.9% (*n* = 26) family participants. 82.2% (*n* = 97) participants indicated that a COVID-19 case had occurred in the nursing home while 17.8% (*n* = 21) had no COVID-19 case occur. Where a COVID-19 case had occurred, participants identified care staff 30.2% (*n* = 26) as the group most impacted, followed by residents 23.2% (*n* = 20), nurses 19.8% (*n* = 17), cleaners 17.4% (*n* = 15) and doctors and relatives 4.7% (*n* = 4) each.

### Experience of residents

The experience of residents was conveyed positively by each of the participant groups: residents, family, and staff (Table 1). The score ranged from 50.9% (*n* = 60) feel safe, to 73.8% (*n* = 87) personal belongings are cared for. All areas scored above 50%, with five questions scoring in the 50% range, six in the 60% range, and two in the 70% range. However, within the high scoring elements the aspects of becoming bored 74 (62.7%), feeling lonely 83 (70.3%) and often feeling worried, anxious, or fearful 75 (63.6%) were scored high. The section Cronbach’s alpha reliability score was strong (0.806).

**Table 1:** Experience of residents

	Strongly Agree / Agree	Don’t Know / Unsure	Disagree / Strongly Disagree
Often become bored	74 (62.7%)	15 (12.7%)	29 (24.5%)
Feel safe	60 (50.9%)	30 (25.4%)	28 (23.8%)
Privacy is respected	69 (58.5%)	20 (16.9%)	29 (24.5%)
Personal belongings are cared for	87 (73.8%)	14 (11.9%)	17 (14.4%)
Can choose which activities to be involved in	67 (56.8%)	16 (13.6%)	35 (29.7%)
Generally, feel happy	70 (59.4%)	13 (11%)	35 (29.7%)
Feel lonely	83 (70.3%)	12 (10.2%)	23 (19.5%)
Staff support to engage in activities that are meaningful	77 (65.2%)	23 (19.5%)	18 (15.2%)
Often feel worried, anxious, or fearful	75 (63.6%)	10 (8.5%)	33 (27.9%)
Content	68 (57.6%)	13 (11%)	37 (31.3%)
Feel listened to by staff	76 (64.4%)	21 (17.8%)	21 (17.8%)
Beliefs and values are accepted by staff	72 (61.0%)	25 (21.2%)	21 (17.8%)
Supported to be independent	78 (66.1%)	10 (8.5%)	30 (25.4%)

### Approach by Staff

The approach of staff was reported positively by the three participant groups, with all areas scoring above 69% (Table 2). Responses ranged from 69.5% (treated fairly by staff) to 83% (treated kindly). All areas scored above 60%, with one question scoring in the 60% range, nine in the 70% range, and three in the 80% range. The section Cronbach’s alpha reliability score was strong (0.973).

**Table 2:** Approach by staff

	Strongly Agree / Agree	Don’t Know / Unsure	Disagree / Strongly Disagree
Treated kindly	98 (83%)	14 (11.9%)	6 (5.1%)
Treated with respect	85 (72.1%)	13 (11%)	20 (16.9%)
Treated fairly by staff	82 (69.5%)	15 (12.7%)	21 (17.8%)
Residents get on well with staff	93 (78.8%)	19 (16.1%)	6 (5.1%)
Can get help from staff when needed	97 (82.2%)	11 (9.3%)	10 (8.5%)
Staff care about residents	91 (77.2%)	20 (16.9%)	7 (5.9%)
Staff explain COVID-19 and its impact in a way that is easy to understand	86 (72.9%)	21 (17.8%)	11 (9.3%)
Can discuss care needs with staff	87 (73.7%)	18 (15.3%)	13 (11%)
Staff treat residents well	96 (81.4%)	14 (11.9%)	8 (6.7%)
Staff support residents with personal care when needed	87 (73.7%)	12 (10.2%)	19 (16.1%)
Feel comfortable speaking to staff about concerns	86 (72.9%)	24 (20.3%)	8 (6.7%)
Staff provide appropriate care based on needs	88 (74.6%)	7 (5.9%)	23 (19.5%)
Staff get on well with residents	92 (78%)	20 (16.9%)	6 (5.1%)

### The nursing home environment

The quantitative data indicates that the nursing home environment was generally viewed positively by participants, with all but one question achieving above 50% (Table 3). The range went from 41.5% (residents get up and go to bed when they want) to 89.1% (nursing home is comfortable and well kept). While one question scored in the 40% range, the remaining questions scored above the 50% range with *n* = 2 in the 50% range, *n* = 12 in the 60%, *n* = 3 in the 70% range and *n* = 2 in the 80% range. The lowest score for agreement was for ‘residents to get up and go to bed when they want’ (41.5%, *n* = 49) with 44.2% (*n* = 52) in disagreement thereby indicating a low level of choice and control. The section Cronbach’s alpha reliability score was strong (0.975).

**Table 3:** The nursing home environment

	Strongly Agree / Agree	Don’t Know / Unsure	Disagree / Strongly Disagree
Nursing home is comfortable and well kept	105 (89.1%)	7 (5.9%)	6 (5%)
Residents get up and go to bed when I want	49 (41.5%)	17 (14.4%)	52 (44.2%)
Residents are encouraged to be part of the community/nursing home	78 (66.2%)	19 (16.1%)	21 (17.8%)
Residents are not discriminated against in any way	64 (54.3%)	30 (25.4%)	24 (20.3%)
Residents are involved in decisions about their care and support	76 (64.4%)	13 (11%)	29 (24.5%)
Residents can choose who else (family, friends) can be involved in their care and support	70 (59.4%)	15 (12.7%)	33 (28%)
Residents can raise concerns and know that they will be dealt with	77 (65.2%)	22 (18.6%)	19 (16.1%)
Residents are happy with the care and support they receive	75 (63.6%)	20 (16.9%)	23 (19.5%)
Residents can get peace and quiet when they want	81 (68.7%)	19 (16.1%)	18 (15.3%)
Residents spend my time doing the things they enjoy	76 (64.4%)	19 (16.1%)	23 (19.5%)
Residents have their room the way they like it	85 (72.1%)	12 (10.2%)	21 (17.8%)
Residents are encouraged to be active	77 (65.2%)	18 (15.3%)	23 (19.5%)
Residents have access to communal spaces to meet fellow residents	100 (84.7%)	5 (4.2%)	13 (11%)
Residents have access to spiritual and religious supports	80 (67.8%)	24 (20.3%)	14 (11.9%)
Residents are supported in keeping contact with family and friends	84 (71.2%)	11 (9.3%)	23 (19.5%)
Residents are supported to use alternative communication means e.g., technology	79 (66.9%)	15 (12.7%)	24 (20.3%)
Residents are supported and given all relevant information to make decisions	77 (65.2%)	17 (14.4%)	24 (20.3%)
Residents can attend online activities such as spiritual services	76 (64.4%)	23 (19.5%)	19 (16.1%)
Residents are supported and given all relevant information regarding advanced care planning	62 (52.5%)	33 (28%)	23 (19.5%)
Residents are accommodated to have visits from family (physical/electronic)	90 (76.3%)	9 (7.6%)	19 (16.1%)

### Open-ended text results

Two specific open-ended questions were asked: ‘If there was a positive case of COVID-19 in your nursing home how did you become aware of this?’ and ‘How did staff support residents to maintain relationships with you as a family and community?’ and these are reported below. In addition, there was an opportunity at the end of the third section to make additional comments and an option for the participant to add anything else about their experience during the COVID-19 pandemic. This additional data was analysed utilising Colarafi and Evans [21] content analysis framework where all qualitative data was collated and read and reread, following which codes were assigned, revised and redefined where appropriate. All codes of similar meaning were grouped together to visualise and represent the data and three themes emerged namely, ‘care’, ‘human rights’ and ‘experiences’.

### If there was a positive case of COVID-19 in your nursing home, how did you become aware of this?

Information regarding positive COVID-19 cases were communicated to staff, residents, and family members in a range of ways. Communication primarily flowed from management teams, directors of nursing, nursing homeowners, or from staff assigned the role of communications. In some cases, staff learned of a positive COVID-19 case from other staff/peers, at staff handover or when coming on duty; *‘I was met at the door and informed (Staff 1)’* which raised a concern for staff who reported *‘you could not always tell what you were walking into (Staff 2)’*.

Several methods were used to support communication, with email being the most frequent. Communication also occurred via text messages, phone calls and encrypted work applications on smartphones. While participants reported communication approaches as effective, they were already established, and there were incidents of residents and family members receiving news about positive COVID-19 cases from other sources; ‘*a relative had to tell me (Resident 1)’* or *‘I hear it when I was down at the local shop (Family member 1)’*. There was a sense among some participants that the presence of COVID-19 was not effectively communicated, *‘we knew COVID-19 was present but we did not know to what was the extent of the outbreak (Resident 2)’*, and that the true reality and seriousness of the situation was masked, *‘I was not told my friend had COVID-19 (fellow resident), I was told he was fine (Resident 3)’*.

### How did staff support residents to maintain relationships with you as a family and community?

A variety of means and measures were identified to assist residents in preserving relationships with family and community. Window visits were frequently employed to facilitate families and friends, whilst also maintaining infection prevention measures and were seen as *‘safe visiting (Family member 2)’*. Visits in outdoor shelters were facilitated as social distancing and outdoor ventilation offered a safer environment for residents. In some cases, families were permitted visits in-person and indoors, while others were only granted in-person visits for compassionate reasons due to residents being unwell or at the end-of-life. Conventional phone calls and/or video calls were a popular means of communication. Staff reported offering forms of technology such as tablets to enable face-to-face communication via WhatsApp and Zoom. Written communications were also supported via postcards, letter writing and emails and where necessary staff aided letter writing and letter reading. One staff participant described using their own personal phone with a resident to support contact with family members. Other endeavours reported to support residents were communicating news updates, reading newspaper, art activities and engaging in essential human interactions such as spending time and sitting to talk and chat with residents.

Resource issues were however evident with family members noting, *‘there is no internet in the nursing home or it’s a poor internet connection (Family member 3)’*, *‘they don’t have phones or tablets and the nursing home only has a few to go around (Family member 4)’*, and in situations where a resident may need assistance to use a phone/tablet, *‘staff did not have much time to help with phone calls they were so busy they were not able to assist at all times (Family member 5)’*. In addition, the demand on staff resulted in family members reporting, *‘being asked to stop ringing the nursing home due to the busyness of the situation (Family member 6)’*, or *‘we had no option to visit, and this was not the case for other nursing homes or the advice at the time (Family member 1)’*. In addition, a family participant reported *‘some residents had more support and visitations than my (family member) and I don’t know why, was there a reason for this or was it that they got favours I don’t know (Family member 8)’*.

### Care

Family members reported their opinions on the level of care their family member received during the peak of the pandemic and associated restrictions. Experiences and perceptions of the care received by residents were expressed along a continuum from excellent to insufficient and neglectful. Family members described their experience as follows, *‘we were in turmoil because we were unable to visit and care for dad’ (Family member 3)*, but there was also an appreciation that residents *‘are in a secure place that they are familiar with staff who care and are doing their best in an unprecedented time’ (Family member 6).* Family members also recognised staffs’ efforts, *‘they did far more than can be expected of them and tried so much to help with everything’ (Family member 4).* Despite families acknowledging the efforts of staff, there was an agreement that there were *‘so little staff to meet resident’s needs’ (Family member 5)*, particularly as *‘staff were sick with COVID-19 themselves and there was no staff to replace them’ (Family member 3)*. Other family members felt care fell short as the holistic needs of residents were not fully addressed as there was a *‘a lack of compassion and understanding as people in the nursing home were lonely and this need was not a priority’ (Family member 9)* and that ‘*people died during COVID-19 alone, confused and without family support and this was so upsetting and cruel’ (Family member 10)*. A family member reported that, *‘if COVID-19 did not cause suffering for people the loneliness and heartbreak experienced during COVID-19 has changed people’s life forever’ (Family member 11)*. There was also a suggestion that staff with suspected COVID-19 infection or contact status continued to work due to staff shortages and a lack of alternative support or options, thereby placing residents at further risk of infection, *‘while we can never know and there was such a shortage of staff you would wonder did staff continue to work because they were needed when if they were working anywhere else they would have been at home as they may have had it’ (family member 12)*.

### Human rights

In the modern healthcare era, there is a greater emphasis on the role and place of human rights and while healthcare may be shaped by human rights, this may not always be evident. Nursing home residents, family members and staff all enjoy rights which were impacted on by various restrictions. Human rights of relevance to this study include the right to life, the right to liberty and security, the right to private and family life, the right to be free from inhuman or degrading treatment, and one’s rights to equality and prohibition of discrimination. Three resident participants reported levels of confusion and distress as *‘it was hard to know who was caring for you with the masks and gowns and it was hard to know what they were saying, all the days were the same and with no contact with the outside world it was confusing well I was getting confused’ (Resident 4)*.

While participants comments may reflect a sense that self-determination, decision-making, and safety were impacted upon, there were also comments that the nursing home was homely *‘it is so welcoming and comfortable in there it’s a real home away from home’ (Resident 5)*. Decision-making and self-determination were perceived as being influenced by the view that, *‘nursing home managers hold all the power, and they were so focused on COVID-19 and it not getting in that they blocked everything and seemed as if they want just to lock the world out till it was over’ (Family member 7)*. There was a sense of hopelessness and powerlessness which stemmed from the feeling of being abandoned (staff and management) or having abandoned their loved ones (families). Here a staff member reported that we have always been forgotten, *‘we never had PPE and there was never any focus on it for nursing homes, but now with COVID-19 nursing homes are in the news everyone is watching, so suddenly we are getting the resources like PPE but why now is it only because of COVID-19 and what will happen after’ (Staff 3)*. From a family perspective not being able to visit their family member especially those with diminished cognitive function or dementia made them question whether their family member felt abandoned, *‘they won’t understand this and what is happening, and I can’t go in and be with them, it’s sad for them to be alone and it’s hard for me thinking that I abandoned them’ (Family member 8)*.

### Experiences

Nursing home staff and families conveyed their opinions on the working situation and their experience during COVID-19. While it was accepted that staff did their best, the toil of COVID-19 was seen as not being without personal and professional cost, *‘staff are so tired and exhausted, they are likely burnt-out but just keep going’ (Family member 9)*. A staff member highlighted that *‘we worked tirelessly to care and support our residents, there were times we worked around the clock and stayed here to keep the residents safe, we made a lot of sacrifices and so did our families and that’s not recognised, my kids had to go without seeing me for a long period of time’ (Staff 4)*. This accumulates in staff considering leaving the nursing home and the caring profession altogether, *‘I have handed in my notice, I will be sad leaving but the whole thing was too much for me I struggled with the restrictions especially for those coming to the end of their life, it played on me and to be honest it was making me unwell so I need to go’ (Staff 5)*. Family members were aware of staffing issues and reported that ‘*there was never enough staff….and they are so busy with little support’ (Family member 10)*.

While participants acknowledged multidisciplinary healthcare team support, it is evident that staff were predominantly nurses or healthcare assistants and that *‘the nurses and carers they are the ones that had to take the brunt of everything and deal with all the stress and pressure from residents, families and the health service’ (Family member 9)*. The need for in-person care provision was also considered essential, *‘the GP (General Practitioner) should have been more supportive they could have done onsite visits instead of over the phone consultations or virtual visits, they were accessible but were not accessible at the same time and its slow to come back’ (Staff 6)*.

In some cases, staff perceived that the regulatory authorities were more concerned with public perception, *‘the regulatory bodies were just responding at times and not to the fore leading and supporting us, they were just regurgitating existing information and to me promoting their presence to ensure they were being heard, we had so many new specialists that I had never heard of before and seemed to lack compassion for people on the ground as people were suffering and they seemed to be focusing on retrospective analysis and surveys information reacting to the crisis but what will we all learn’ (Staff 6)*. This response highlights that regulatory bodies, professors, researchers, health authorities and professionals were at the forefront of media reporting around nursing homes, providing advice and guidance. However, nursing was less evident, and a staff member deemed the cohort in the media as *‘out of touch with the daily reality of nursing homes and the lives of people living and working there’ (Staff 7)*. Nonetheless, there was a sense that the ‘media circus’ would help shine a light on issues affecting nursing homes including personal protective equipment availability, but also emphasise the *‘reality of the sadness, fear, anxiety and individual tragedies that occurred and force us to question if they were warranted and necessary’ (Staff 8)*. This culminated in staff wavering between a feeling that support would finally be on its way and the feeling that they would be forgotten again as support would dispel once they were out of the media.

## Discussion

The results of this study highlight both the positive and negative aspects of care provision in the participating nursing homes. It is evident from this and other studies that nursing home facilities during the COVID-19 pandemic faced uncertainty and changing restrictions that impacted on nursing home residents, staff and family members [22]. Restrictions implemented because of the COVID-19 pandemic limited people’s ability to engage in group activities and led to a corralling of residents and staff (same shift and work/living areas), less mixing and engagement within the nursing home, visiting restrictions, and the limiting or stopping of contact with outside facilitators or activities [23]. While these were essential limitations, they did impact on residents’ sense of loneliness and isolation [24, 25]. The findings of this study and others highlight that the sense of isolation and loneliness is not an unusual aspect of the COVID-19 nursing home experience [26, 27]. The issue of restriction, lack of visits and staff wearing unfamiliar personal protective equipment emphasised the sense of loneliness and isolation, and it was not seen as the appropriate form of action in all cases especially where residents reportedly died alone [28]. While lower scoring elements and negative comments were evident in the open-ended questions, such as those related to maintaining communication, these must be considered in the context of staff confronting, on a daily basis, the ever shifting and changeable landscape of what to expect when going on duty [29, 30].

Difficulties were described for nursing homes and their staff in ensuring they were up to date with advice and guidance issued by the government and regulatory bodies. This was fuelled by a flow of new information resulting in ever changing guidance and policy updates, creating a divergence in interpretation and in implementation [31]. Such variations or time lags concerning what was believed allowable and safe across different nursing homes, irrespective of public health advice is a concern and a lesson we must learn from for the future in relation to communication strategies and one source of information for all. This notion of one source of information and developing communication strategies is promoted in the literature [32, 33]. Where differences in communication exist, it may be due to organisations defining their own rules, operating outside of or behind government guidelines, and ultimately results in the inconsistent protection of human rights [34–37].

The results of this study suggest that participants were generally happy with communication concerning COVID-19, but they also emphasise the continuous need for effective and inclusive communication along with the availability of technological resources [38, 39]. The favourable evaluation of communication in this study may represent the adequate tailoring of messages to foster an inclusive communication environment and the addressing of vulnerabilities that may place an unnecessary burden, e.g., higher infection and death rates, on this cohort as compared with others in the context of public health crises [40]. A scoping review by Veiga-Seijo et al. [41] highlights effective strategies such as: the use of virtual communication platforms (such as video calls, phone calls, and messaging apps); providing training and support to residents and families to use these tools; promoting safe outdoor visits and window visits that comply with infection control guidelines, allowing residents to safely interact with their families in person; implementing structured visitor programs that allow family members to schedule and plan visits while adhering to safety protocols; providing regular updates to families about their loved ones’ well-being, activities, and any changes in care plans via phone calls, emails, or newsletters; offering emotional support services and counselling for both residents and families to address feelings of isolation, anxiety, and loneliness; organising virtual or socially distanced activities that involve families, such as virtual games, storytelling sessions, or remote celebrations for birthdays and holidays; partnering with community organisations and volunteers to facilitate family connections, such as delivering care packages, coordinating outdoor visits, or providing technology support; implementing flexible visitation policies that can adapt to changing circumstances and public health guidelines, ensuring that family connections remain a priority while maintaining safety; training nursing home staff to prioritise and facilitate family connections, including communication skills, empathy training, and infection control measures and advocating for policy changes and resources at the local and national levels to support meaningful family connections in nursing homes during public health emergencies. Situations arose where residents were not able to use technology, poor or no internet reception existed, or were only available in communal spaces which were now unavailable to residents. These were significant issues, and all greatly impacted on communication for residents with family and the world outside the nursing home [41, 42]. Other studies reported difficulties such as that options like window visits were only possible for residents on ground floors which created frustration for families who then wanted their loved one moved [43, 44].

The commitment of staff to go beyond their anticipated role to assist residents in their care was reported by participants in this study. This was evident in nursing staff activities such as supporting and promoting communication, for instance, through assistance with phone or video calls as well as written correspondence including letters and email. This emphasises the support provided and the need to value these activities as reflected in the wider literature [23, 45] and where efforts may have fallen short, this appeared to be related to the demands and pressures placed on staff in particular staffing levels and staff shortages [46–48]. While staffing levels could have potentially been an issue due to illness and work demands there was no specific questions as to workload or staffing in this study and thereby no evidence of this occurrence within this study. In the wider literature the evidence is mixed, Werner and Coe [49] identify that nursing home staffing levels did not significantly change during COVID-19 while Shen et al. [50] highlight that outbreaks were related to a statistically significant drop in nursing staffing levels due to raised absences and departures. However, the real issues may be the difficult choices nursing home managers were confronted with in the early stages of COVID-19 as they tried in extraordinary times to offset infection control, safety issues, rights, and humanity issues [51, 52]. As all concerned (nursing homes, government, and society) learned more about COVID-19 and the advent of vaccinations such restrictions relaxed.

It is important to acknowledge the rights of residents in nursing homes and their families. While an ethical dilemma existed in the sense of keeping most people safe, there was certainly an interference with individual’s human rights such as their right to self-determination. Under Article 8(2) of the European Convention on Human Rights [53] such a restriction may be justified provided it is in accordance with law and proportionate to the aims to be achieved. While a detailed assessment of proportionality is beyond the aims and scope of this paper it can be noted that the imposition of restrictions should not be done in such a way as to exacerbate underlying vulnerabilities. A study by Anand et al. [54] investigated the impact of the COVID-19 pandemic on nursing (care) homes across seven countries in Europe, highlighting significant issues such as deaths, damage, and violations of human rights leading to severe consequences for residents, including high mortality rates and neglect. The relationship between palliative care and rights holds significance in terms of access to quality palliative care which is crucial for ensuring that older people receive adequate support, symptom management, and comfort at the end of life. However, during the pandemic, there were reported deficiencies in providing palliative care services due to overwhelmed healthcare systems, limited resources, and the isolation measures in place [55].

For frontline staff, findings of this and other studies describe a fearful and traumatic experience [47, 56–58]. Participants internationally describe how the value and contribution of their service to their patients was largely unrecognised [47, 59]. Their separation from their own families at the height of COVID-19 to protect the residents in their care and their additional working hours received scant acknowledgment [60]. Staff participants highlighted the fact that some were left burntout and demoralised at what they had or were witnessing and left the service [61, 62]. The constraints imposed on older people coming to the end of their life was too much for some staff to witness and resulted in resignation [63, 64]. The need to successfully react to a pandemic has raised the importance of securing the wellbeing and safety of the most vulnerable in society, and this study relays the hope that lessons realised will inform practice and policy in the future.

The COVID-19 pandemic created extraordinary challenges across all facets of society. It represented not only a public health crisis but also a crucial juncture for the respect and safeguarding of human rights. In such circumstances, human rights and assurances of equality are of great importance to protect against an erosion of societal values and to maintain and protect social norms. In this regard, the pandemic raised serious questions about how we protect the right to life, the right to liberty and security, the right to respect for private and family life, and the prohibition of discrimination, in addition to the concept of human dignity. It is therefore not surprising that it has been labelled as ‘a defining moment for human rights’ [65]. Residential care settings have a crucial contribution to make in this respect, as they deliver care to a group of people who are recognised as some of the most vulnerable, in environments that are highly predisposed to infection transmission. It was not within the scope of this article to determine whether specific rights were infringed, instead it concentrated on points of weakness as well as strength in the protection of human rights. Some of these points are closely tied to COVID-19 while others inevitably reflect legacy issues which persist across the Irish healthcare system. It follows that the lessons learned must not only inform future epidemic and pandemic planning but must also shape our response to areas of enduring weakness so that human rights can be fully realised at all stages of care and treatment.

### Strengths and limitations

A strength of this study was that the study addresses a critical and timely issue related to the impact of the COVID-19 pandemic on nursing homes, providing insights that can inform public health responses and policies. Secondly, utilising a mixed-methods approach, combining quantitative survey data with qualitative insights offered a richer understanding of the complex issues involved. Thirdly, collaboration with key stakeholders using a steering group enhanced the development of the questionnaire and applicability of the study’s findings. Finally, the integration of data both qualitative and quantitative offers a more nuanced understanding of the complexities surrounding nursing homes response during the COVID-19 pandemic. However, the results must also be considered in terms of the limitations and several limitations exist. Firstly, a sampling bias may exist given that a limited number of nursing homes in Ireland participated in this study. Secondly, the use of predefined questions may have limited the depth of information obtained and while open-ended questions were used, they are less scalable for large-scale surveys. Thirdly, survey research cannot establish causation between variables. Fourthly, low response rates may introduce non-response bias and the sample size in this study was low.

## Conclusion

This study sought to capture the experience and views of residents, families, and staff in nursing homes, and this is important as respect for human rights should advise and shape the development of national policy documents and public health guidance. The perspectives of nursing home residents, their families, and staff should be included in public health advisory groups to inform the development of future epidemic/pandemic planning strategies. Evident from this study is that nursing homes’ communication plans and tools need to be improved to ensure communications are delivered in a timely and suitable manner. Furthermore, nursing homes need to be supported in altering, creating and applying additional resources to encourage communication for residents, such as, upgrade of internet connections and purchasing tablet computers. A key factor arising from this study is that palliative care should have a more central role in national epidemic/pandemic planning documents to ensure a more grounded reaction to infectious disease outbreaks. In addition, nursing homes should have better support and clarity in applying policy at a local level so as discrepancies regarding what is deemed permitted and safe across different nursing home settings do not exist. These aspects are important as nursing home residents are generally older people in their later years of life and there needs to be a focus on quality of life in these later years and to ensure we protect their vulnerability. However, while healthcare professionals have a duty to uphold the rights of nursing home residents, they themselves have human rights which must also be protected and supported.

### Electronic supplementary material

Below is the link to the electronic supplementary material.

Supplementary Material 1

Supplementary Material 2

## Data Availability

The datasets used and/or analysed during the current study are available from the corresponding author on reasonable request.
