# Supplementary material for: The palliative care experience in Irish nursing homes during the COVID-19 pandemic: a survey of residents, family, and staff
Source: BMC Palliat Care. 2024 May 22;23:126. doi: 10.1186/s12904-024-01458-8 (PMC11110399; doi:10.1186/s12904-024-01458-8)
Supplement: Supplementary file 1 — Supplementary Material 1 [file 12904_2024_1458_MOESM1_ESM.docx]

**Supplementary file 1: Survey questionnaire***

**Shaping Palliative Care Policy using a Human Rights-Based Approach**

**Section One – Person**

*For all questions, please tick the appropriate box on the rating scale as follows*:

Strongly Agree; Agree; Don’t know or Unsure; Disagree; Strongly Disagree

| **Question** | | **Strongly**  **Agree**  **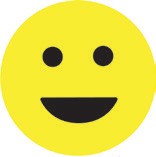** | **Agree**  **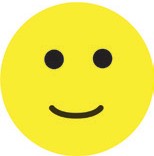** | **Don’t Know / Unsure**  **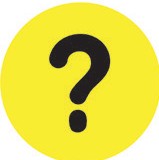** | **Disagree**  **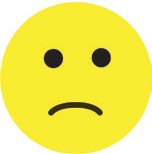** | **Strongly Disagree**  **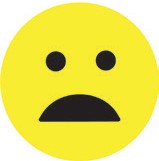** | **Prefer not to answer**  **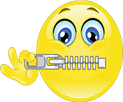** |
| --- | --- | --- | --- | --- | --- | --- | --- |
| **The following statements describe my experience in this nursing home since the start of the COVID-19 pandemic:** | | | | | | | |
| Q.1 | My family member often become bored | 🞎 | 🞎 | 🞎 | 🞎 | 🞎 | 🞎 |
| Q.2 | My family member feels safe | 🞎 | 🞎 | 🞎 | 🞎 | 🞎 | 🞎 |
| Q.3 | My family member privacy is respected | 🞎 | 🞎 | 🞎 | 🞎 | 🞎 | 🞎 |
| Q.4 | My family member belongings are cared for | 🞎 | 🞎 | 🞎 | 🞎 | 🞎 | 🞎 |
| Q.5 | My family member can choose which activities I get involved in | 🞎 | 🞎 | 🞎 | 🞎 | 🞎 | 🞎 |
| Q.6 | My family member generally feels happy | 🞎 | 🞎 | 🞎 | 🞎 | 🞎 | 🞎 |
| Q.7 | My family member feels lonely | 🞎 | 🞎 | 🞎 | 🞎 | 🞎 | 🞎 |
| Q.8 | My family member is supported to engage in activities that are meaningful to them | 🞎 | 🞎 | 🞎 | 🞎 | 🞎 | 🞎 |
| Q.9 | My family member often feel worried, anxious, or fearful | 🞎 | 🞎 | 🞎 | 🞎 | 🞎 | 🞎 |
| Q.10 | My family member is content | 🞎 | 🞎 | 🞎 | 🞎 | 🞎 | 🞎 |
| Q.11 | My family member is listened to | 🞎 | 🞎 | 🞎 | 🞎 | 🞎 | 🞎 |
| Q.12 | My family member beliefs and values are accepted | 🞎 | 🞎 | 🞎 | 🞎 | 🞎 | 🞎 |
| Q.14 | My family member is supported to be independent | 🞎 | 🞎 | 🞎 | 🞎 | 🞎 | 🞎 |
| Any comments | | | | | | | |

**Section Two – Staff**

*For all questions, please tick the appropriate box on the rating scale as follows*:

Strongly Agree; Agree; Don’t know or Unsure; Disagree; Strongly Disagree

| **Question** | | **Strongly**  **Agree**  **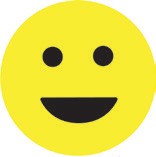** | **Agree**  **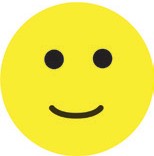** | **Don’t Know / Unsure**  **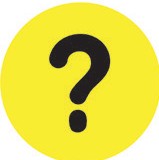** | **Disagree**  **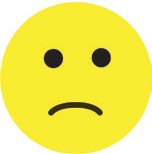** | **Strongly Disagree**  **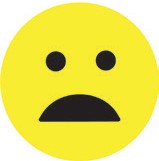** | **Prefer not to answer**  **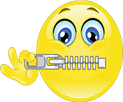** |
| --- | --- | --- | --- | --- | --- | --- | --- |
| **The following statements describe my experience in this nursing home since the start of the COVID-19 pandemic:** | | | | | | | |
| Q.15 | My family member is treated kindly | 🞎 | 🞎 | 🞎 | 🞎 | 🞎 | 🞎 |
| Q.16 | My family member treated with respect | 🞎 | 🞎 | 🞎 | 🞎 | 🞎 | 🞎 |
| Q.17 | My family member is treated fairly | 🞎 | 🞎 | 🞎 | 🞎 | 🞎 | 🞎 |
| Q.18 | My family member gets on well with staff | 🞎 | 🞎 | 🞎 | 🞎 | 🞎 | 🞎 |
| Q.19 | Staff help My family member when they need assistance | 🞎 | 🞎 | 🞎 | 🞎 | 🞎 | 🞎 |
| Q.20 | Staff care about my family member | 🞎 | 🞎 | 🞎 | 🞎 | 🞎 | 🞎 |
| Q.21 | Staff explain COVID and its impact in a way that is easy to understand | 🞎 | 🞎 | 🞎 | 🞎 | 🞎 | 🞎 |
| Q.22 | Staff discuss care needs with my family member | 🞎 | 🞎 | 🞎 | 🞎 | 🞎 | 🞎 |
| Q.23 | Staff treat my family member well | 🞎 | 🞎 | 🞎 | 🞎 | 🞎 | 🞎 |
| Q.24 | Staff support my family member with personal care when needed | 🞎 | 🞎 | 🞎 | 🞎 | 🞎 | 🞎 |
| Q.25 | Staff are comfortable speaking to my family member about their concerns | 🞎 | 🞎 | 🞎 | 🞎 | 🞎 | 🞎 |
| Q.26 | Staff provide appropriate care based on my family member needs | 🞎 | 🞎 | 🞎 | 🞎 | 🞎 | 🞎 |
| Q.27 | Staff get on well with my family member | 🞎 | 🞎 | 🞎 | 🞎 | 🞎 | 🞎 |
| Any comments | | | | | | | |

**Section Three – Service**

*For all questions, please tick the appropriate box on the rating scale as follows*:

Strongly Agree; Agree; Don’t know or Unsure; Disagree; Strongly Disagree

| **Question** | | **Strongly**  **Agree**  **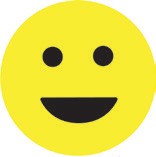** | **Agree**  **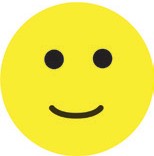** | **Don’t Know / Unsure**  **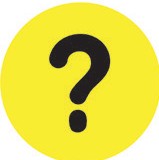** | **Disagree**  **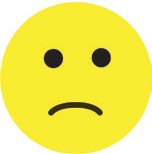** | **Strongly Disagree**  **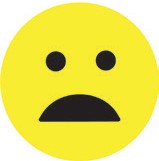** | **Prefer not to answer**  **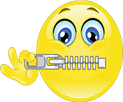** |
| --- | --- | --- | --- | --- | --- | --- | --- |
| **The following statements describe my experience in this nursing home since the start of the COVID-19 pandemic:** | | | | | | | |
| Q.28 | The nursing home is comfortable and well kept | 🞎 | 🞎 | 🞎 | 🞎 | 🞎 | 🞎 |
| Q.29 | Residents get up and go to bed when they wish | 🞎 | 🞎 | 🞎 | 🞎 | 🞎 | 🞎 |
| Q.30 | Residents are encouraged to be part of the community/nursing home | 🞎 | 🞎 | 🞎 | 🞎 | 🞎 | 🞎 |
| Q.31 | Residents are not discriminated against in any way | 🞎 | 🞎 | 🞎 | 🞎 | 🞎 | 🞎 |
| Q.32 | Residents are involved in decisions about their care and support | 🞎 | 🞎 | 🞎 | 🞎 | 🞎 | 🞎 |
| Q.33 | Residents can choose who else (family, friends) is involved in their care and support | 🞎 | 🞎 | 🞎 | 🞎 | 🞎 | 🞎 |
| Q.34 | Residents can raise concerns and know that they will be dealt with | 🞎 | 🞎 | 🞎 | 🞎 | 🞎 | 🞎 |
| Q.35 | Residents are happy with the care and support they receive | 🞎 | 🞎 | 🞎 | 🞎 | 🞎 | 🞎 |
| Q.36 | Residents can get peace and quiet when they want | 🞎 | 🞎 | 🞎 | 🞎 | 🞎 | 🞎 |
| Q.37 | Residents can spend time doing the things they enjoy | 🞎 | 🞎 | 🞎 | 🞎 | 🞎 | 🞎 |
| Q.38 | Residents have their room the way they like it | 🞎 | 🞎 | 🞎 | 🞎 | 🞎 | 🞎 |
| Q.39 | Residents are encouraged to be active | 🞎 | 🞎 | 🞎 | 🞎 | 🞎 | 🞎 |
| Q.40 | Residents have access to communal spaces to meet fellow residents | 🞎 | 🞎 | 🞎 | 🞎 | 🞎 | 🞎 |
| Q.41 | Residents have access to spiritual and religious supports | 🞎 | 🞎 | 🞎 | 🞎 | 🞎 | 🞎 |
| Q.42 | Residents are supported in keeping contact with family and friends | 🞎 | 🞎 | 🞎 | 🞎 | 🞎 | 🞎 |
| Q.43 | Residents are supported to use alternative communication means e.g., technology | 🞎 | 🞎 | 🞎 | 🞎 | 🞎 | 🞎 |
| Q.44 | Residents are supported and given all relevant information to make decisions | 🞎 | 🞎 | 🞎 | 🞎 | 🞎 | 🞎 |
| Q45 | Residents can attend online activities such as spiritual services | 🞎 | 🞎 | 🞎 | 🞎 | 🞎 | 🞎 |
| Q.46 | Residents are supported and given all relevant information regarding advanced care planning | 🞎 | 🞎 | 🞎 | 🞎 | 🞎 | 🞎 |
| Q.47 | Residents are accommodated to have visits from family (physical / electronic) | 🞎 | 🞎 | 🞎 | 🞎 | 🞎 | 🞎 |
| Any comments | | | | | | | |

**Section Four – Demographic Information**

**Q.48 Age** ____________________

**Q.49 Gender** _________________

**Q.50 How long have you been a staff in the nursing home?** ___________

**Q.51 Have there been any COVID-19 cases in your nursing home?**

**Yes** 🞎  **No** 🞎

**Q.51A If yes, were the people who tested positive for COVID-19 in your nursing home:** (Please tick as many as appropriate)

**Other residents** 🞎 **Relatives** 🞎 **Care staff** 🞎 **Nurses** 🞎 **Cleaners** 🞎 **Doctors** 🞎

**Q.51B If there was a positive case of COVID-19 in your nursing home how did you become aware of this?** ______________________________________________________________________

______________________________________________________________________

______________________________________________________________________

______________________________________________________________________

______________________________________________________________________

**Q.52 Was information provided about COVID clear and understandable?**

**Yes** 🞎  **No** 🞎

**Q.53 How did you support residents maintain relationships with family and community?** ______________________________________________________________________

______________________________________________________________________

______________________________________________________________________

______________________________________________________________________

______________________________________________________________________

______________________________________________________________________

**Q.54 Is there anything else you would like to add about your experience during the COVID-19 pandemic?**

______________________________________________________________________

______________________________________________________________________

______________________________________________________________________

______________________________________________________________________

______________________________________________________________________

______________________________________________________________________

* Wording of questionnaire altered to target respondents three version one for nursing home resident, one for family and one for staff of the nursing home.
